# The Need for Translational Research to Advance Peripheral Artery Disease Management

**DOI:** 10.3390/ijms160511125

**Published:** 2015-05-18

**Authors:** Joseph V. Moxon, Jonathan Golledge

**Affiliations:** 1 The Queensland Research Centre for Peripheral Vascular Disease, James Cook University, Townsville, QLD 4811, Australia; E-Mail: joseph.moxon@jcu.edu.au; 2 Department of Vascular and Endovascular Surgery, the Townsville Hospital, Townsville, QLD 4814, Australia

Peripheral artery disease (PAD) is a broad term encompassing a range of atherosclerotic and aneurysmal conditions of the extra-coronary arteries [1]. PAD is estimated to affect up to 15% of the population aged over 65 years, and is predicted to become increasingly common in parallel with an ageing population [2]. Indeed, recent data suggest that global abdominal aortic aneurysm (AAA) related mortality increased by ~10% between 1990 and 2010 [3]. Similarly, the world-wide prevalence of lower limb atherothrombosis increased by ~20% in the 10 years between 2000 and 2010 [4]. There are significant shortfalls in current PAD management, including a need for better ways to diagnose PAD early and a requirement for new treatment options. PAD is recognized to present a cardiovascular risk equivalent to coronary heart disease, although risk factor management appears to be less intensive in PAD patients, possibly due to a primary treatment focus on the presenting complaint [5,6,7,8,9]. Thus, there is a clear need to improve PAD patient care through directed research. We are delighted to present a Special Issue of the *International Journal of Molecular Sciences* [10] which includes 11 articles focused on PAD. Collectively these articles cover aspects of AAA and lower limb atherothrombosis and discuss findings of current research using human tissues and animal models. We are extremely grateful to all contributing authors and peer-reviewers for their excellent work and support in making this Special Issue possible.

Addressing the shortfalls in PAD management remains problematic due to our incomplete understanding of disease pathophysiology. Limited access to arterial biopsies from patients and suitable controls presents a significant hurdle to PAD research [11]. This has in part been addressed through the development of rodent models for PAD which have been widely employed in the literature, although their relevance and translational potential to the clinical situation is not always immediately clear [12]. This is highlighted by Lin and colleagues who provide an excellent overview of widely used PAD models, and discuss the role of imaging techniques to provide data on PAD formation and progression in experimental animals [13]. Such imaging modalities are under-utilised in preclinical PAD research, and the authors point out that their potential to provide novel insight of *in vivo* pathology is largely untapped. In line with the sentiment of this Special Issue, Lin and colleagues emphasise that effective translation of research from small animals requires parallel advances in our understanding of PAD pathogenesis in both animal models and human patients.

Six of the papers in this Special Issue focus on AAA which still presents a significant scientific and clinical challenge, arguably due to its multifactorial nature. Inflammation and matrix remodeling are strongly implicated in AAA [2]. A large amount of pre-clinical data has suggested that doxycycline through its ability to inhibit matrix metalloproteinases and inflammation might have a therapeutic value for AAA [14]. In this Special Issue Kroon and Taanman discuss the potential of doxycycline as a treatment for AAA and explore potential anti-inflammatory mechanisms by which this drug may limit AAA growth [15]. A recent large clinical trial failed to demonstrate any value of doxycycline in limiting AAA growth [16]. Kroon and Taanman argue that this could be due to the use of too low a dose of the drug although the dose used was based on prior testing in patients [17]. A further trial is on-going and the results of this study are expected to resolve whether doxycycline has any value in AAA patients (detailed at https://clinicaltrials.gov/ct2/show/NCT01756833). Data presented by Hinterseher *et al.* [18] and Yoshimura *et al.* [19] provide further insight into the relevance of the inflammatory process in AAA pathology. The study by Hinterseher builds on prior genomic data and investigates in detail the association of the natural killer cell-mediated cytotoxicity pathway with AAA presence [18]. Similarly, Yoshimura and colleagues present data to support a role for statins in suppressing the inflammatory process within the AAA wall [19]. Although the association of dyslipidaemia with AAA remains controversial [1], findings from Yoshimura and colleagues hint at a potential benefit of statin therapy for AAA patients beyond traditional lipid lowering roles.

A positive family history increases AAA risk ~2-fold, suggesting that genetic factors are important in AAA development [20,21]. Genetic and bioinformatic investigations by Pahl *et al.* identify changes in the binding properties of several transcription factors in AAA patients compared to controls [22]. Similarly Ryer and colleagues report alterations to the circulating DNA methylation profile in AAA patients compared to controls [23]. This is further supported by Spear *et al.* who report that the micro-RNA expression profile in AAA-associated tissues is also reflected in patient plasma and can differentiate AAA patients from controls [24]. Collectively, these data suggest that alterations in gene regulation may be important determinants of AAA and may provide tools to screen at-risk populations.

This Special Issue also contains a mix of publications examining biochemical and patient-oriented strategies to improve the management of lower-limb atherothrombosis. Lower limb atherothrombosis is the most common form of PAD and it is widely recognised that improved management strategies are needed for patients that present with this problem [25]. Improvements are needed in a number of areas, including earlier diagnosis, more effective and varied treatment options, and better adherence to evidence-based medicine. The work presented in this issue illustrates some of the current challenges in researching lower limb atherothrombosis. Krishna and colleagues describe some of the pathophysiological aspects of lower limb atherothrombosis. The authors focus particularly on circulating biomarkers which could theoretically be brought into clinical practice to facilitate early diagnosis, better target current therapies and monitor response to treatment [26]. While Krishna *et al.* illustrate that many circulating markers have been associated with PAD presence, severity, and outcome, none have been shown to guide management exclusively for patients with lower limb atherothrombosis. One pathophysiological mechanism implicated in the complications of lower limb atherothrombosis by Krishna and colleagues is oxidative stress. Hernandez-Aguilera and colleagues investigate the expression of a group of antioxidant enzymes called paraoxonases (PON) [27]. They report that expression of PON1 and PON3 is upregulated within the arteries and blood of patients with lower limb atherothrombosis. They also link the expression of PON with the up-regulation of the chemokine CCL2. They suggest that PON and its related chemokines could be involved in the pathogenesis of atherosclerosis within the lower limb but whether this association is causal remains to be established.

Supervised exercise is an established treatment for patients with lower limb atherothrombosis presenting with intermittent claudication [28]. Leicht and colleagues examine the effect of supervised exercise on anthropometric measures in patients with intermittent claudication [29]. They report that patients randomised to supervised exercise experience a smaller increase in body fat compared to patients undertaking medical management alone. This was observed despite there being no change in dietary pattern amongst the patients receiving supervised exercise. The findings are further evidence for the value of exercise in patients with intermittent claudication. Finally, Je and colleagues report the use of the Peripheral Artery Questionnaire (PAQ) in Korean patients with lower limb atherothrombosis [30]. This health-related quality of life tool is one of few which has been validated for use in Korean patients with lower limb atherothrombosis. The investigators examined a group of patients that had undergone revascularisation and report that improvements in the PAQ responses were correlated with improvements in the ankle-brachial pressure index (ABPI). ABPI is an established tool for assessing the response to revascularisation [31]. The investigators also demonstrate that there are some patients in which there are disparate changes in the PAQ and ABPI after revascularisation. It is likely that both measures have independent value in assessing the outcomes of interventions to treat lower limb atherothrombosis.

Overall the studies included in this Special Issue illustrate some of the unique features and challenges of PAD which require tailored solutions. Many of the treatments currently applied in PAD patients have been adopted from findings in other fields, particularly cardiology. There is an on-going need for research specifically focused on PAD in order to address the current requirements for improved diagnostics and treatments for this important and growing medical problem. The multifactorial nature of PAD, and the presence of multiple co-morbidities in patients likely presents the most significant hurdle to realizing this goal. It is our opinion that successful translation of findings in the short-to-medium term will be best achieved by focusing on the end-users (*i.e.*, the patient and clinician), as opposed to generating data solely of biological interest. For example, recent advances in molecular and biochemical techniques permit elaborate analyses which yield large biological datasets, but are typically limited to relatively small sample sizes due to technical and financial restrictions. In such studies, identifying relevant outcomes from complex data can be challenging, and the generalizability of findings to the wider PAD patient population is not immediately apparent when these approaches are applied in isolation [12,32]. We therefore strongly believe that multi-disciplinary studies collaboratively conducted by laboratory scientists, clinicians and patients are vital to advance our understanding, and ultimately the management of PAD.
